# Which Factors Influence Teacher Report of Adaptive Functioning in Autistic Children?

**DOI:** 10.1007/s10803-021-04930-z

**Published:** 2021-03-12

**Authors:** Heather L. Moore, Leanne Rogan, Lauren J. Taylor, Tony Charman, Ann Le Couteur, Jonathan Green, Victoria Grahame, the PACT-G Consortium, Catherine Aldred, Matea Balabanovska, Hilary Beach, Claire Bennett, Sophie Carruthers, Imogen Crook, Hannah Danvers, Kate Dartnall, Ceri Ellis, Richard Emsley, Hannah Foote, Jessica Graham, Patricia Howlin, Neil Humphrey, Kirsty James, Sarah Jamieson, Anna Kappa, Anna Knight, Kathy Leadbitter, Jo Lowe, Sophie Langhorne, Ruth Madeley, Deborah Maskell, Olivia Mitchell, Helen McConachie, Francisca Monteiro, Cat Papastavrou Brooks, Jeremy Parr, Amelia Pearson, Andrew Pickles, Jessica Rose, Lisa Slater, Vicky Slonims, Carol Taylor, Susanna Vosper, Helen Wilson

**Affiliations:** 1 School of Psychology, Newcastle University, Dame Margaret Barbour Building, Wallace Street, Newcastle upon Tyne, NE2 4DR UK; 2 Cumbria, Northumberland, Tyne and Wear NHS Foundation Trust, Walkergate Park, Benfield Rd, Newcastle upon Tyne, NE6 4QD UK; 3 Leeds Teaching Hospitals Trust, St James University Hospital, Beckett Street, Leeds, LS9 7TF UK; 4 University of Leeds, Worsley Building, Clarendon Way, Woodhouse, Leeds, LS2 9NU UK; 5 Department of Psychology, Institute of Psychiatry, Psychology & Neuroscience, King’s College London, Henry Wellcome Building, De Crespigny Park, Denmark Hill, Box PO77, London, SE5 8AF UK; 6 Present Address: School of Psychological Science, University of Western Australia, 35 Stirling Highway, Crawley, WA 6009 Australia; 7 Population Health Sciences Institute, Level 3, Sir James Spence Institute, Royal Victoria Infirmary, Newcastle University, Queen Victoria Road, Newcastle upon Tyne, NE1 4LP UK; 8 Division of Neuroscience and Experimental Psychology, University of Manchester, PACT-G Trial Office, Room 3.312, Jean McFarlane Building, Oxford Road, Manchester, M13 9PL UK; 9 Royal Manchester Children’s Hospital, Manchester University NHS Foundation Trust, Oxford Road, Manchester, M13 9WL UK

**Keywords:** Autism, Adaptive functioning, Behaviour, Teacher report

## Abstract

A wealth of parent-report research shows adaptive functioning difficulties in autistic children, with parent-report influenced by a number of child factors. Adaptive functioning in autistic children is known to vary across settings; however, no research has yet explored factors influencing education professional-report. This study investigated the rate and profile of impairment, and child factors influencing education professional-reported adaptive skills in 248 autistic children. Twelve children were < 3 years (min age for available normative data on the adaptive function measure), so were removed from the analyses. Results replicated parent-literature; adaptive skills were negatively associated with age and informant-reported autism severity, and positively associated with nonverbal ability and expressive language. Adaptive functioning is important for real-world outcomes, e.g. educational attainment, independence, and support needs. Improving our understanding of adaptive functioning in the education context may support opportunities for shared learning and enhance personalised support .

**Supplementary Information:**

The online version of this article (10.1007/s10803-021-04930-z) contains supplementary material, which is available to authorized users.

Adaptive functioning describes practical, everyday skills required to meet the demands of the environment. Such difficulties are commonly reported in autistic individuals (Maskey et al. 2012) and link to real-world outcomes such as educational attainment, likelihood of independent living, and requirement for support services (De Bildt et al. 2005; Farley et al. 2009; Taylor and Henninger 2015). Longitudinal follow up of autistic adults shows low rates of independent living, employment, friendships and romantic relationships (Magiati et al. 2014), highlighting the importance of understanding adaptive skills profiles in autistic children for developing personalised support, to improve long term outcomes.

Most research assessing autistic children has used parent-report Vineland Adaptive Behaviour Scales [VABS; (Sparrow et al. 1984a, b, 2005)], comprising Communication, Daily Living Skills (DLS), Socialisation, Motor Skills, and an overall Adaptive Composite Score (ABC). This informant-report measure utilises information from people who know an individual well and have observed their skills in one or more everyday settings (Merrell 2000), which is especially important for autistic children, who may struggle to communicate, and may lack insight into their own difficulties. Parent-report research broadly reports relative strengths in Motor Skills and relative weaknesses in Socialisation and/or Communication domains (e.g. Nevill et al. 2017; Yang et al. 2016). Significantly lower levels of adaptive functioning are reported by parents of autistic children compared to control groups matched for chronological age (CA) and/or development/IQ, and other neurodevelopmental disorders (e.g. Mouga et al. 2015; Paul et al. 2014; Ventola et al. 2014).

Positive associations are consistently identified between cognitive ability and adaptive functioning in autistic children (Klin et al. 2007; Nevill et al. 2017), although relative to measured IQ, low functioning children show strengths in adaptive ability and high functioning children show weaknesses (e.g. Alvares et al. 2020; Tillmann et al. 2019), weakening the explanatory power of cognitive ability in this group. Few studies have considered language ability independently of cognition; those that did have identified significant positive correlations between language and adaptive skills (e.g. Mayo et al. 2013; Di Rezze et al. 2019). Cross-sectional research indicates a negative relationship between age and adaptive functioning in autistic children (e.g. Klin et al. 2007; McDonald et al. 2015; Nevill et al. 2017). Longitudinal studies, however, reveal a more complex picture of change and stability, influenced by factors such as verbal and nonverbal ability (Farmer et al. 2018; Paynter et al. 2018; Szatmari et al. 2015; Lord et al. 2015). Associations between adaptive functioning and autism severity using direct assessment are variable, with some showing no relationship (Nevill et al. 2017; Ray-Subramanian et al. 2011; Yang et al. 2016), while others identified small but significant negative associations (Paul et al. 2014; Green and Carter 2014; Kanne et al. 2011). In contrast, using parent-report consistently establishes negative relationships (Duncan and Bishop 2015; Liss et al. 2001; McDonald et al. 2015; Perry et al. 2009). Two studies have reported a negative relationship between adaptive functioning and child behaviour problems (Gillham et al. 2000; Green and Carter 2014).

Adaptive functioning in autistic children has been shown to vary over time and across context (Ozonoff et al. 2005; McDonald et al. 2016; De Los Reyes 2011). Thus, it may be helpful to gain additional information from different contexts, for example, in the education setting. To date, little research has explored the factors influencing teacher-reported adaptive functioning in autistic children. Four studies have investigated concordance between parent- and teacher-reported adaptive functioning with varying results (Dickson et al. 2018; Lane et al. 2013; McDonald et al. 2016; Jordan et al. 2019); however, none of these studies investigated which factors influence teacher-report. As adaptive functioning is known to influence educational attainment (De Bildt et al. 2005; Brady et al. 1992), it is important to understand which factors affect teacher-reported adaptive functioning of autistic children.

In order to address this knowledge gap, we used the baseline data of the Paediatric Autism Communication Trial-Generalised (PACT-G; see Green et al. (2018), for details of the trial protocol) to investigate the teacher-report version of the VABS-II (T-VABS-II) in a sample of autistic children. The aims of this study were to determine rates of impairment, compare domain scores for relative strengths and weaknesses in performance, and explore whether any child factors predict reporting of adaptive behaviour on the T-VABS-II. Guided by the parent-report adaptive functioning literature, we hypothesised that:

1. Children younger than 7 years (sub-scale age cut-off) would show relative strengths in T-VABS-II Motor Skills. Differences in other domains were explored for the whole sample.
2. CA, nonverbal and language ability, and informant-report of autism severity would show a significant relationship with T-VABS-II ABC and domain scores.

We also explored the role of teacher-reported behavioural difficulties on T-VABS-II scores.

## Methods

### Participants

Baseline data were used from 248 children, aged 2–11 years, who were recruited to PACT-G (Green et al. 2018) between November 2016 and April 2018. PACT-G is a randomised controlled trial of a social communication intervention for autistic children. Children in Greater Manchester, the North East of England, and South London were recruited via referral from local clinical and educational services. Eligible children had a clinical diagnosis of autism, which was confirmed using Autism Diagnostic Observation Schedule-2 (ADOS-2; Lord et al. 2012) cut-off scores for ‘Autism’ and Social Communication Questionnaire (SCQ) scores of ≥ 12 for children < 5 years or ≥ 15 for children ≥ 5 years (Rutter et al. 2003). All children had nonverbal age-equivalent (AE) scores of > 12 months, measured using the Visual Reception (VR) and Fine Motor (FM) subscales of the Mullen Scales of Early Learning (MSEL; Mullen 1995), or the Special Nonverbal Composite Score on the British Ability Scales-School Age (BAS; Elliott and Smith 2011). Children ≥ 5 years were between P3 and P8 on the English curriculum.^1^ Children with controlled epilepsy were included. Children/parents with significant hearing/visual impairments were excluded, as were parents with severe learning disability or psychiatric disorder. See Table 1 for child characteristics. Parents required enough spoken/written English to participate in PACT-G assessments and intervention. A favourable ethical opinion was obtained from the North West-Greater Manchester Central Research Ethics Committee (REF: 15/NW/0912). Parents provided informed, written consent, and the education provider agreed to participate (see Table 1 for school type).

**Table 1:** Demographic characteristics of PACT-G sample (N = 248)

	N (%)	Min	Max	Mean	SD
Child CA (months)	248 (100)	26	131	61.10	22.36
Child Gender					
Female	51 (21)				
Male	197 (79)				
Child ethnicity					
White-British	136 (55)				
White non-British	13 (5)				
Mixed/Multiple ethnic backgrounds^a^	23 (9)				
Asian/Asian-British	30 (12)				
Black/African/Caribbean/Black British	40 (16)				
Other ethnic group^b^	6 (2)				
Type of school					
Mainstream nursery	94 (38)				
Specialist nursery	5 (2)				
Mainstream primary school	50 (20)				
Mainstream school with SEN/autism resource class	8 (3)				
Special school with mixed disabilities	55 (22)				
Specialist autism school	35 (14)				
Childminder	1 (1)				
Phrase speech (ADOS-2 Module^c^)					
Module 1	187 (75)				
Module 2	61 (25)				

^a^Includes mixed white and black Caribbean, white and black African, white and Asian, and any other mixed backgrounds

^b^Includes Arab

^c^ADOS-2 module 1: nonverbal-simple phrases e.g. two words); ADOS-2 module 2: short phrases upwards

### Measures

Characterisation data from the PACT-G sample used in this study included^2^:

#### Adaptive Functioning

The T-VABS-II (Sparrow et al. 2005) is a teacher-assessed questionnaire of adaptive ability for ages 3–21 years. Raw scores were translated into standard scores for Communication, DLS, Socialisation, Motor Skills, and ABC, with a mean of 100 and SD of 15.

#### Autism Severity

The ADOS-2 (Lord et al. 2012) is a semi-structured, play-based assessment of social communication and restricted and repetitive behaviours. Calibrated Severity Scores (CSS) from 1 to 10 were calculated, which are standardised in relation to CA and verbal ability.

The SCQ Lifetime (Rutter et al. 2003) is a 40-item, parent-report questionnaire that measures social communication behaviours relevant to autism.

#### Non-verbal Ability

The VR and FM subscales from the MSEL (Mullen 1995) measure nonverbal ability. As our sample included children > 5 years (outside the age range to derive standard scores), we calculated a nonverbal developmental quotient (NVDQ; see Statistical Analysis).

#### Language Ability

Receptive and Expressive One Word Picture Vocabulary Test (ROWT, EOWT; Martin and Brownell 2011a, b) are picture-based assessments of understanding and use of single words. We used raw scores to capture performance variation of all participants, including those who did not score sufficient correct responses to derive a t-score.

#### Child Behaviour

The Teacher Strength and Difficulties Questionnaire (T-SDQ; Goodman 1997) measures emotional, conduct, hyperactivity/inattention, peer relationships and prosocial behaviour.

### Procedure

Assessments administered directly with the child took place at the research clinic, child’s home and/or education setting. Questionnaires were provided to parents and education professionals to complete during or between sessions and return to the research team. Education settings chose the most appropriate person to complete questionnaires, based on prior knowledge of the child. Both parents and education staff were given opportunities to ask questions about any items prior to submission.

### Statistical Analysis

Data were prepared and analysed using IBM SPSS Statistics Version 24 (Corp 2016). Twelve participants were removed from the analyses as they were < 3 years, thus younger than the youngest available normative data for the T-VABS-II. Table 2 shows N values for each variable included in the analyses. Teacher-report measures (T-VABS-II and T-SDQ) were defined as missing if education professionals did not return questionnaires or returned them in an incomplete fashion, e.g. T-VABS-II: insufficient subscale items completed to calculate domain scores, or if participants were older than the CA subscale cut-off of 7+ years for T-VABS-II Motor Skills; T-SDQ: < 60% of items completed. Of researcher-administered measures, two participants completed the BAS (Elliott and Smith 2011) so did not have MSEL NVDQ scores. Heightened distress meant it was not possible to complete the ROWT and EOWT with a small number of participants. SDQ subscale scores were prorated according to hand-scoring instructions (Goodman 2001). We used nonparametric equivalents for any data that were not normally distributed.

To determine rates of impairment on T-VABS-II, we examined the percentage of individuals whose ABC and domain standard scores were > 2SD below the normative mean (i.e. < 70). We used a repeated measures ANOVA to compare T-VABS-II domain scores.

Based on findings from the parent VABS literature, we undertook a series of multiple linear regression analyses to investigate whether there were concurrent associations between T-VABS-II ABC/domain scores and child CA, autism severity, nonverbal ability, language ability, and child behaviour, which was supported by correlations between variables in our own data (Supplementary Table 1). All models were examined to ensure that they did not violate the assumptions of linear regression, including multicollinearity (using the VIF). While ADOS-2 CSS did not correlate with T-VABS-II standard scores, we included it to explore prior variable reports regarding direct assessments of autism severity. There were no significant differences between MSEL VR and FM AE scores (Z = − 0.13, p = 0.900; Mean MSEL VR = 27.43 months, Mean MSEL FM = 27.24 months) so we used a mean score to calculate the MSEL NVDQ (nonverbal mental AE/CA*100). Participants performed significantly better on the ROWT than EOWT (Z = − 4.55, p < 0.001); therefore, we entered these predictors separately, rather than deriving a language quotient.

## Results

Descriptive statistics (including N) for each measure are presented in Table 2. While the range of T-VABS-II ABC and domain scores showed that some individuals were within AE levels, mean performance in each area was markedly lower than age expectations. Of participants with T-VABS-II ABC scores, 78% scored more than 2SD below the norm. For domain scores, this equated to 69% for Communication, 67% for DLS, 77% for Socialisation, and 50% for Motor Skills. A within subjects ANOVA to compare T-VABS-II domains, using Greenhouse–Geisser correction, indicated significant differences [F(2.78, 503.62) = 13.88, p < 0.001]. Pairwise comparisons showed that participants performed better on the Motor Skills than Communication (p = 0.002), DLS (p = 0.001), and Socialisation (p < 0.001) domains, but no other domains differed significantly. When removing DLS to increase sample size, the nonsignificant difference between other domains remained [F(1.89, 426.06) = 0.38. p = 0.675].

**Table 2:** Descriptive statistics for measures of autism severity, adaptive functioning, language ability, strengths and difficulties, and nonverbal ability

	N	Min	Max	Mean	SD
T-VABS-II ABC Standard Score	221	25	98	58.04	14.86
T-VABS-II Communication Standard Score	227	27	114	61.88	16.78
T-VABS-II DLS Standard Score	228	23	108	61.39	16.29
T-VABS-II Socialisation Standard Score	229	23	93	61.43	11.73
T-VABS-II Motor Skills Standard Score	185	38	103	68.61	13.11
ADOS CSS	236	6	10	7.40	1.26
SCQ Total Score	236	12	36	23.58	5.23
MSEL NVDQ	234	12.60	112.24	48.06	18.79
ROWT Total Score	235	0	80	18.75	22.26
EOWT Total Score	232	0	85	15.78	19.23
T-SDQ Total Score	224	5	30	17.51	4.64

Regression analyses for T-VABS-II ABC and domain scores are reported in Table 3. Child CA, parent-report SCQ, and T-SDQ were significant negative predictors of T-VABS-II ABC, while MSEL NVDQ and EOWT were significant positive predictors, accounting for 73% of the variance. Significant negative predictors of T-VABS-II Communication were Child CA, SCQ, and T-SDQ, and significant positive predictors were MSEL NVDQ, ROWT and EOWT, accounting for 77% of the variance. T-VABS-II DLS were significantly negatively associated with Child CA, SCQ, and T-SDQ, but positively associated with MSEL NVDQ and EOWT, accounting for 70% of the variance. Significant negative predictors of T-VABS-II Socialisation were Child CA, SCQ, and T-SDQ, and significant positive predictors were MSEL NVDQ and EOWT, accounting for 58% of the variance. Finally, T-VABS-II Motor Skills scores were square root transformed due to non-normal unstandardised residuals [Shapiro–Wilk(176) = 0.99, p = 0.049]. Motor Skills were significantly negatively associated with Child CA and SCQ, and significantly positively associated with ADOS-2 CSS and MSEL NVDQ, accounting for 29% of the variance.

**Table 3:** Regression models for T-VABS-II adaptive ability, using child age, autism severity, nonverbal ability, language ability and strengths and difficulties as predictors

	B (CI)	SE B	β	p	Adjusted r^2^
T-VABS-II ABC					0.73
(Constant)	63.38 (50.80, 76.00)	6.38		< .001**	
Child CA	− 0.21 (− 0.28, − 0.14)	0.04	− 0.32	< .001**	
ADOS-2 CSS	0.30 (− 0.55, 1.15)	0.43	0.03	.484	
SCQ	− 0.26 (− 0.48, − 0.05)	0.11	− 0.09	.015*	
MSEL NVDQ	0.26 (0.16, 0.37)	0.05	0.35	< .001**	
ROWT	0.07 (− 0.04, 0.18)	0.06	0.11	.206	
EOWT	0.20 (0.09, 0.32)	0.06	0.28	.001**	
T-SDQ	− 0.34 (− 0.58, − 0.10)	0.12	− 0.11	.006*	
T-VABS-II Communication					0.77
(Constant)	57.80 (44.92, 70.68)	6.53		< .001**	
Child CA	− 0.18 (− 0.26, − 0.11)	0.04	− 0.25	< .001**	
ADOS-2 CSS	0.74 (− 0.14, 1.61)	0.44	0.06	.099	
SCQ	− 0.24 (− 0.45, − 0.02)	0.11	− 0.08	.035*	
MSEL NVDQ	0.31 (0.20, 0.41)	0.05	0.36	< .001**	
ROWT	0.15 (− 0.04, 0.26)	0.06	0.20	.008*	
EOWT	0.24 (0.11, 0.36)	0.06	0.28	< .001**	
T-SDQ	− 0.33 (− 0.58, − 0.08)	0.13	− 0.10	.009*	
T-VABS-II Daily Living Skills					0.70
(Constant)	79.23 (34.56, 94.01)	7.47		< .001**	
Child CA	− 0.28 (− 0.36, − 0.19)	0.04	− 0.38	< .001**	
ADOS-2 CSS	− 0.38 (− 1.37, 0.61)	0.50	− 0.03	.453	
SCQ	− 0.32 (− 0.56, − 0.07)	0.13	− 0.10	.013*	
MSEL NVDQ	0.31 (0.19, 0.42)	0.06	0.36	< .001**	
ROWT	− 0.02 (− 0.14, 0.11)	0.06	− 0.02	.817	
EOWT	0.23 (0.09, 0.36)	0.07	0.27	.001**	
T-SDQ	− 0.46 (− 0.74, − 0.18)	0.14	− 0.13	.001**	
T-VABS-II Socialisation					0.58
(Constant)	80.63 (68.41, 92.85)	6.20		< .001**	
Child CA	− 0.11 (− 0.18, − 0.04)	0.04	− 0.22	.002**	
ADOS-2 CSS	− 0.58 (− 1.41, 0.26)	0.42	− 0.06	.173	
SCQ	− 0.35 (− 0.56, − 0.15)	0.10	− 0.16	.001**	
MSEL NVDQ	0.13 (0.03, 0.23)	0.05	0.22	.009**	
ROWT	− 0.03 (− 0.13, 0.08)	0.05	− 0.05	.613	
EOWT	0.25 (0.14, 0.37)	0.06	0.43	< .001**	
T-SDQ	− 0.53 (− 0.76, − 0.30)	0.12	− 0.22	< .001**	
T-VABS-II Motor Skills (sq root transformed)					0.29
(Constant)	7.86 (6.63, 9.08	0.62		< .001**	
Child CA	− 0.01 (− 0.02, 0.00)	0.01	− 0.18	.039*	
ADOS-2 CSS	0.08 (0.03, 0.16)	0.04	0.14	.043*	
SCQ	− 0.03 (− 0.05, − 0.01)	0.01	− 0.20	.002**	
MSEL NVDQ	0.01 (0.01, 0.02)	0.01	0.33	.004**	
ROWT	0.01 (− 0.00, 0.02)	0.01	0.24	.087	
EOWT	− 0.00 (− 0.02, 0.01)	0.01	− 0.01	.465	
T-SDQ	0.01 (− 0.01, 0.03)	0.01	0.07	.351	

*Significant at .05 level

**Significant at .01 level

## Discussion

As far as the authors are aware, this is the first study to investigate education professional reporting of adaptive functioning in autistic children. We saw high rates of impairment, with at least 50% of children in the ‘low’ range on T-VABS-II ABC/domains (> 2SD below norm). Consistent with our hypotheses, we found relative strengths in Motor Skills, and teacher-report was significantly associated with child factors such as CA, parent-report autism severity, teacher-report behaviour problems, nonverbal and language ability.

Our higher relative T-VABS-II Motor Skills performance is consistent with previous parent-report findings (e.g. Nevill et al. 2017; Yang et al. 2016). In contrast though, we did not find relative weaknesses in T-VABS-II Socialisation or Communication domains. Instead, we found a relatively flat profile with consistent low performance (excluding Motor Skills). This finding may relate to our sampling design, which included older children with lower cognitive abilities that may have negatively impacted adaptive functioning.

Turning to factors that influence T-VABS-II report, nonverbal ability positively predicted ABC/domain scores, in accordance with previous parent-report research (e.g. Perry et al. 2009; Nevill et al. 2017). Unsurprisingly, when looking specifically at the role of language, both receptive and expressive language positively predicted T-VABS-II Communication domain. Additionally, expressive language predicted ABC, DLS and Socialisation. Expressive language may show a stronger relationship to adaptive functioning because many of the T-VABS-II items rely on observation of use or lack of expressive skills, whereas appreciation of language understanding may not be captured as readily. Our results extend previous associations found in the parent literature (e.g. Mayo et al. 2013; Di Rezze et al. 2019) by exploring receptive and expressive language independently.

Child CA and parent-reported autism severity were significant negative predictors of adaptive functioning across all T-VABS-II scores, consistent with parent-report VABS literature (Saulnier and Klin 2007; Klin et al. 2007; McDonald et al. 2015; Nevill et al. 2017). While our data is cross-sectional, so it is not possible to make conclusions about longitudinal, age-related changes, it is likely that autistic children continue to develop their adaptive abilities over time. However, they may not progress at the same rate as typically developing peers, leading to a steady deviation from the age-expected developmental trajectory.

The contribution of direct child autism severity assessment to parent-reported adaptive functioning is inconsistent (e.g. Nevill et al. 2017; Paul et al. 2014), although parent-reported measures have identified significant negative relationships (e.g. Duncan and Bishop 2015; McDonald et al. 2015). However, informant-reported autism severity in parent literature may bias perceptions of adaptive functioning. We dissociated ratings of autism severity (parent SCQ) and adaptive functioning (T-VABS-II) to reduce this risk of observer bias, and identified negative associations with adaptive functioning. There was no relationship with direct assessment of autism severity (ADOS-2), which could be expected given the variable parent literature, except for a positive association with Motor Skills. This relationship was unexpected, especially given the high prevalence of motor difficulties in autism (Green et al. 2009), and further research is needed to elucidate this finding.

These differences might be explained by the properties of the measures. The ADOS-2 is undertaken during a set period and as such, may not capture all autism-related features across different contexts that are more broadly relevant to adaptive behaviour. Further, the ADOS-2 CSS may not necessarily be sensitive enough to tease out differences in autism severity between individuals. Conversely, items on the SCQ may overlap with the T-VABS-II, creating stronger associations as a result. Informant measures tend also to record the most severe behaviour exhibited by a child, so in some cases, these scores may represent early deficits or extremes of behaviour rather than current symptoms or general presentation. It is possible, though, that these early deficits may have a lasting impact on development of adaptive abilities, explaining the variability between these two types of measure.

Finally, our study found reduced adaptive functioning with increasing T-SDQ difficulties in all T-VABS-II scores except Motor Skills, supporting previous findings (Gillham et al. 2000; Green and Carter 2014), although some variance may be explained by the similarity in themes of items on both measures. Autistic children have high rates of emotional and behavioural problems (Maskey et al. 2012; Chandler et al. 2016); thus, it is particularly relevant to explore further how this impacts on everyday functioning.

Our results show that similar child characteristics influence education professional and parent report of adaptive functioning (as identified extensively in previous parent literature). Children with lower adaptive abilities require greater special education provision (De Bildt et al. 2005), suggesting this may be an important area of focus in education. However, autistic children can have difficulties generalising learnt behaviours across different contexts (McDonald et al. 2016); thus, differences may arise in parent and education professional reports of adaptive functioning. While a small number of studies have shown moderate agreement in parent- and teacher-reported domain scores (Dickson et al. 2018; Lane et al. 2013; McDonald et al. 2016; Jordan et al. 2019), no study to date has examined whether this is driven by consistent responses to individual behaviours within each domain. To better understand adaptive functioning across contexts, we plan to undertake a detailed comparison of parent and teacher VABS-II item level responses (Moore et al. in preparation).

Our results are potentially limited by lack of knowledge about the background and experience of the education professionals who completed our questionnaires. Informants differed by level of education and teaching experience, as well as autism training and experience, type of education environment, class size and potentially, familiarity with the child. The T-VABS-II assumes knowledge about developmental levels and terminology that may vary based on informant characteristics. It is not possible for the researchers to understand whether informants used standard thresholds for scoring within each item in this study, but in future, it would be beneficial to capture more detailed information about informant characteristics and factor these into analyses, to determine any impact on reporting.

Our findings indicate real adaptive functioning difficulties as reported by education professionals, and influences of CA, informant-reported autism severity, nonverbal ability and expressive language on reporting. Adaptive skills are important for real-world outcomes of autistic individuals, including educational attainment, level of support needs, and independence, making them an important educational focus. Gaining further understanding of the relationships between adaptive skills and other skills may enhance opportunities for shared learning and inform personalised support, to improve long term outcomes.

## Supplementary Information

Below is the link to the electronic supplementary material.

Electronic supplementary material 1 (DOCX 17 kb)

## Data Availability

Access to PACT-G data will be available in due course subject to consideration by the PACT-G Consortium and current NIHR guidance.

## Footnotes

1 In England, P scales describe targets for children aged 5–16 years with special educational needs. P8 was taken to represent a language age-equivalent of approximately 4 years in a typically developing child.

2 Data from selected measures were used for the purpose of this study. For complete baseline characterisation data of the PACT-G sample, see Green et al. (2018).
