# Supplementary material for: Which Factors Influence Teacher Report of Adaptive Functioning in Autistic Children?
Source: J Autism Dev Disord. 2021 Mar 12;52(1):463–72. doi: 10.1007/s10803-021-04930-z (PMC8732898; doi:10.1007/s10803-021-04930-z)
Supplement: Supplementary file 1 — Electronic supplementary material 1 (DOCX 17 kb) [file 10803_2021_4930_MOESM1_ESM.docx]

*Supplementary Table 1.* Spearman Correlations between child characteristics and T-VABS-II scores.

|  |  | 1 | 2 | 3 | 4 | 5 | 6 | 7 | 8 | 9 | 10 | 11 |
| --- | --- | --- | --- | --- | --- | --- | --- | --- | --- | --- | --- | --- |
| 1 | T-VABS-II ABC Standard Score |  |  |  |  |  |  |  |  |  |  |  |
| 2 | T-VABS-II Communication Standard Score | .929^**^ |  |  |  |  |  |  |  |  |  |  |
| 3 | T-VABS-II DLS Standard Score | .919^**^ | .826^**^ |  |  |  |  |  |  |  |  |  |
| 4 | T-VABS-II Socialisation Standard Score | .834^**^ | .703^**^ | .719^**^ |  |  |  |  |  |  |  |  |
| 5 | T-VABS-II Motor skills Standard Score | .741^**^ | .589^**^ | .626^**^ | .460^**^ |  |  |  |  |  |  |  |
| 6 | Child CA | -.538^**^ | -.412^**^ | -.613^**^ | -.373^**^ | -.410^**^ |  |  |  |  |  |  |
| 7 | ADOS-2 CSS | -.025 | -.018 | -.028 | -.112 | .094 | -.068 |  |  |  |  |  |
| 8 | SCQ Total Score | -.316^**^ | -.224^**^ | -.289^**^ | -.284^**^ | -.184^**^ | .277^**^ | .071 |  |  |  |  |
| 9 | MSEL NVDQ | .803^**^ | .798^**^ | .764^**^ | .620^**^ | .533^**^ | -.558^**^ | -.111 | -.250^**^ |  |  |  |
| 10 | ROWT Total Score | .546^**^ | .634^**^ | .413^**^ | .478^**^ | .307^**^ | .096 | -.181^**^ | -.042 | .563^**^ |  |  |
| 11 | EOWT Total Score | .573^**^ | .667^**^ | .450^**^ | .513^**^ | .291^**^ | .082 | -.073 | .000 | .545^**^ | .860^**^ |  |
| 12 | T-SDQ Total Score | -.275^**^ | -.284^**^ | -.261^**^ | -.328^**^ | -.070 | -.028 | .117 | .074 | -.314^**^ | -.289^**^ | -.250^**^ |

*Note:* *: Correlation is significant at .05 level, **: Correlation is significant at .01 level
